# Mast Pulses Shape Trophic Interactions between Fluctuating Rodent Populations in a Primeval Forest

**DOI:** 10.1371/journal.pone.0051267

**Published:** 2012-12-10

**Authors:** Nuria Selva, Keith A. Hobson, Ainara Cortés-Avizanda, Andrzej Zalewski, José Antonio Donázar

**Affiliations:** 1 Institute of Nature Conservation, Polish Academy of Sciences, Kraków, Poland; 2 Environment Canada, Saskatoon, Saskatchewan, Canada; 3 Department of Conservation Biology, Estación Biológica de Doñana, Consejo Superior de Investigaciones Científicas, Sevilla, Spain; 4 Mammal Research Institute, Polish Academy of Sciences, Białowieża, Poland; University of California, Berkeley, United States of America

## Abstract

How different functional responses of consumers exploiting pulsed resources affect community dynamics is an ongoing question in ecology. Tree masting is a common resource pulse in terrestrial ecosystems that can drive rodent population cycles. Using stable isotope (δ^13^C, δ^15^N) analyses, we investigated the dietary response of two fluctuating rodent species, the yellow-necked mouse *Apodemus flavicollis* and the bank vole *Myodes glareolus*, to mast events in Białowieża Forest (NE Poland). Rodent hair samples were obtained non-invasively from faeces of their predators for an 11-year period that encompassed two mast events. Spectacular seed crops of deciduous trees, namely oak *Quercus robur* and hornbeam *Carpinus betulus*, occur after several intermediate years of moderate seed production, with a post-mast year characterised by a nil crop. While a Bayesian isotopic (SIAR) mixing model showed a variety of potential vegetation inputs to rodent diets, the isotopic niche of the yellow-necked mouse was strongly associated with mast of deciduous trees (>80% of diet), showing no variation among years of different seed crop. However, bank voles showed a strong functional response; in mast years the vole shifted its diet from herbs in deciduous forest (∼66% of diet) to mast (∼74%). Only in mast years did the isotopic niche of both rodent species overlap. Previous research showed that bank voles, subordinate and more generalist than mice, showed higher fluctuations in numbers in response to masting. This study provides unique data on the functional response of key pulse consumers in forest food webs, and contributes to our understanding of rodent population fluctuations and the mechanisms governing pulse–consumer interactions.

## Introduction

Resource pulses, defined as brief and infrequent events of high resource availability, are widespread phenomena in nature that have tremendous impacts on consumer communities, including bottom-up effects with consequences for multiple trophic levels [1]–[3]. One of the most common types of pulsed resources in terrestrial ecosystems is mast seeding, especially among temperate tree species [1]. Masting is the synchronous intermittent production of large seed crops by a population of plants [4]. It occurs after several intermediate years of moderate seed production, and is followed by a post-mast year often characterised by a nil crop. In deciduous forests, masting triggers rapid growth in rodent densities which is typically followed by a population crash, thus initiating a sequence of effects which propagate throughout the food web [1], [5]–[8]. How resource pulses elicit different responses of consumers and how this, in turn, affects community dynamics through competitive interactions are ongoing questions in ecology [2].

Pulsed resources affect consumer demography and resource use [1], [2]. Besides food availability, trophic interactions, parasites and predation have been shown to also play a role in rodent population dynamics [6], [9], [10]. In particular, the role of interspecific competition in shaping the structure and dynamics of rodent communities is of considerable interest [11], [12]. Niche divergence or resource partitioning are a key mechanism that can reduce conflicts among similar species with overlapping niche requirements. In this sense, niche overlap is often interpreted in light of potential competition [13]–[16]. However, documenting competition in natural communities is difficult due to complexities associated with past competition and challenges related to the quantification of resource limitation and their use by consumers at the individual and population level [12], [13], [16], [17]. Masting events provide a natural experiment in resource manipulation to investigate how resource pulses mediate competitive and trophic interactions among consumer species. Although the numerical response of consumers to pulses has been widely documented [1], [2], few studies have additionally included evidence of these consumers using the pulsed resource [18].

Here, we used a stable isotope approach to investigate the dietary response of two sympatric and ecologically similar rodent species to mast cycles (consisting of mast, post-mast and intermediate years) in the Białowieża primeval forest (NE Poland). This approach allowed us to avoid some of the shortcomings of tracing nutrient flow through animal communities using conventional dietary analysis techniques [19]. The isotope approach is particularly amenable when the pulsed resource differs isotopically from other available foods [20], [21] and when exploring the generalized ecological niche of co-occurring individuals and populations [22].

Our main objective was to investigate trophic segregation between two rodents, the bank vole *Myodes glareolus* and the yellow-necked mouse *Apodemus flavicollis*, by tracing the consequences of superabundant food pulses provided by mast events. Both species follow population fluctuations triggered by masting; their numerical response has been well documented [5], [6], [23]. We hypothesized that masting will cause significant changes in the stable isotope (δ^13^C, δ^15^N) composition of both species. According to ecological theory [16], [17], during mast events we expected a dietary and isotopic convergence of the two species. We anticipated that if interspecific competition for food resources was important, during non-mast years the species would differ isotopically due to trophic or dietary segregation. We expected a functional response of both species to mast cycles, with larger variation in the food niche of bank voles, the smaller and subordinate species [12], [24].

## Materials and Methods

### (a) Ethics Statement

All necessary permits were obtained for the described field studies. Samples did not belong to any protected species. The Białowieża National Park administration issued the permit for the collection of vegetation samples in this protected area; no permit was required for sample collection in the commercial unprotected part of the forest. The field studies did not involve endangered or protected species. The Mammal Research Institute in Białowieża gave permit to access its collection; rodent specimens were loaned to collect hair samples.

### (b) Study Area

Białowieża Forest is a well-preserved lowland temperate forest located in NE Poland, at the Polish-Belarusian border. The most primeval part is the Strict Reserve of the National Park, dominated by oak- lime-hornbeam (*Quercus robur*-*Tilia cordata- Carpinus betulus*) forest [6]. Spectacular and synchronized seed crops of oak and hornbeam occur at 6–9 year intervals, followed by a year of nil crop, and moderate crops in the intervening years. In the autumn of mast years, the biomass of seeds produced by pristine oak-lime-hornbeam stands can be up to almost 2000 kg/ha, in comparison to c.a. 300 kg/ha of seeds produced on average in intermediate years [5]. The community of forest rodents, dominated by bank voles and yellow-necked mice, represents a main mast consumer. Their populations peak in autumn of the year after the mast (post-mast year) and crash afterwards [5], [6].

### (c) Sample Collection

Plant samples (N = 161, representing 42 species or groups, Table S1) were collected in 2007–2011 in an area of about 160 km^2^, including the Strict Reserve of the Białowieża Forest, the most primeval, and its surrounding area. We collected fruits and seeds from different individual plants, mainly trees, and, in the case of herbaceous vegetation, leaves from those species most abundant and representative of the main habitat/forest types (deciduous forest, coniferous forest, alderwoods and meadows).

Rodent hair samples (N = 110) were obtained non-invasively from the remains of predator faeces collected during previous diet studies in the primeval part of the forest [25] and stored at the museum of the Mammal Research Institute in Białowieża. Hair samples for isotope analysis were selected to maximize the representation of mast, post-mast and intermediate years, and of adult individuals. We focused on an 11-year period (1986–1996) that encompassed the oak and hornbeam masting of autumn 1989 and 1995 [5], [6]. All samples belonged to the period October-April, which corresponded to rodent hair grown in autumn. Hair samples were classified as corresponding to “mast” years, “post-mast” years (the year following the mast, with nil crop), and “intermediate” years of moderate seed production (neither mast nor post-mast year).

### (d) Stable Isotope Analysis

Plant material was washed in distilled water, dried, and powdered prior to isotope analysis. Hair was first rinsed in a 2∶1 chloroform:methanol solution to remove surface oils and cut into small pieces. Samples were combusted in a Eurovector 3000 (Milan, Italy–http://www.eurovector.it) elemental analyzer. The resulting CO_2_ and N_2_ analyte gas from the samples was separated by Gas Chromatograph (GC) and introduced into a Nu Horizon (Nu Instruments, Wrexham, UK–http://www.nu-ins.com) triple-collector isotope-ratio mass-spectrometer via an open split and compared to a pure CO_2_ or N_2_ reference gas. For hair, the Bowhead Whale baleen standard BWB II (δ^13^C = −18.5‰, δ^15^N = 14.4‰) and porcine (PRC) gelatin (δ^13^C = −13.5‰, δ^15^N = 4.69‰) laboratory standards were used. For plant material, we used an-house peagrain standard (δ^13^C = −24.7‰, δ^15^N = 2.4‰). Measurement errors based on within-run measurements of standards (SD) were better than ±0.2‰. All stable isotope ratios were reported in the delta notation as parts per thousand (‰) deviation from an appropriate international elemental standard according to the equation:
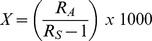
where X is the heavy isotope (^13^C, ^15^N), R_A_ is the isotopic ratio (^13^C/^12^C, ^15^N/^14^N) of the sample and R_S_ is the isotope ratio of the international standard (δ^13^C: Vienna Pee Dee Belemnite carbonate (VPDB); δ^15^N: atmospheric nitrogen (AIR)).

### (e) Statistical Analysis

**Figure 1:**
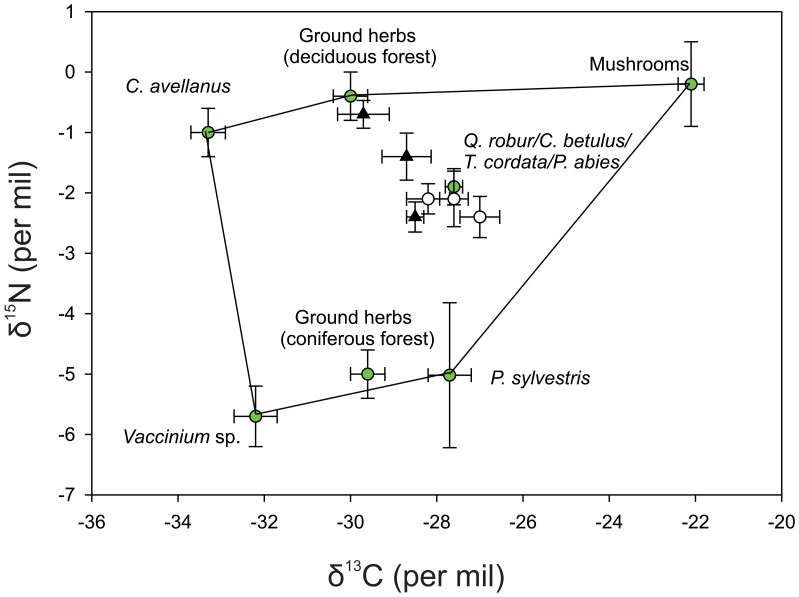
Convex mixing polygon (excluding lichens) used to model potential rodent dietary inputs. Vegetation groups (green circles) correspond to those listed in Tables 1 and S2. Triangles are bank voles and circles are yellow-necked mice (see Figure 2 for corresponding intermediate, mast and post-mast categories depicted).

We were interested in whether or not mast from individual tree species could be identified isotopically in order to trace mast pulses through small-mammal communities. Our a priori assumption was that plant communities would segregate according to the four main habitat/forest types in Białowieża Forest [6]: (a) deciduous-dominated forest (oak-lime-hornbeam forest); (b) coniferous-dominated forest, composed mainly of Norway spruce *Picea abies* and Scots pine *Pinus silvestris*, (c) alderwoods and streamside forest, dominated by black alder *Alnus glutinosa* and (d) meadows, with sedges, grasses and forbs. Spruce admixtures are common in all forest types. We used MANOVA and a Tukey post-hoc analyses to test among vegetation categories. Plant samples represented 42 species or groups (Table S1). From these isotopic analyses we considered 18 vegetation categories relevant to rodent diets (F_17,136_ = 11.7, p<0.001). Tukey’s post-hoc tests identified 4 homogeneous subsets for δ^15^N and 5 subsets for δ^13^C. These corresponded to 8 vegetation groups which could be distinguished based on at least one isotope (Table 1). Those vegetation groups were portrayed (excluding lichens) in bivariate (δ^13^C and δ^15^N) space in preparation for a mixing model analysis (Figure 1). The contributions of isotopically distinct dietary endpoints to the diets of the two species were then investigated further using the Bayesian mixing model SIAR [26] (Table S2) in R.2.10.1.

**Figure 2:**
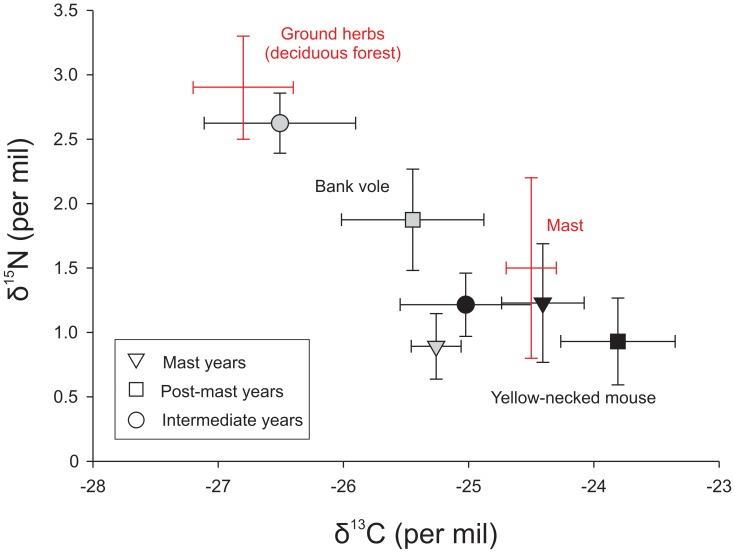
Isotope values of the rodent species along mast cycles. Mean (± S.E.) δ^15^N and δ^13^C values for bank voles (grey symbols) and yellow-necked mice (black symbols) in mast, post-mast and intermediate years. Also shown are positions animals would occupy if they had exclusive diets of deciduous forest herb vegetation and mast seeds (oak, hornbeam, lime, spruce).

**Table 1:** Isotopically distinct vegetation groupings (mean±SE) used to examine dietary niches of the two rodent species in Białowieża Forest.

Vegetation Group	N	δ^13^C (‰)	δ^15^N (‰)
**Ground herbs:**			
1. Coniferous forest^a^	5	−29.6±0.4	−5.0±0.3
2. Deciduous forest^b^, alderwoods^c^, meadow graminoids	23	**−**30.0±0.4	**−**0.4±0.4
**Shrubs:**			
3. *Vaccinium* sp.	6	**−**32.2±0.5	**−**5.7±0.5
**Trees:**			
4. *Pinus silvestris*	6	**−**27.7±0.5	**−**5.0±1.2
5. *Corylus avellana*	6	**−**33.3±0.4	**−**0.8±0.4
6. *Quercus robur/Carpinus betulus/Tilia cordata/Picea abies*	31	**−**27.7±0.2	**−**1.8±0.7
**Other:**			
7. Mushrooms	6	**−**22.1±0.3	**−**0.22±0.7
8. Lichens	1*	**−**26.0	**−**12.0

Sample sizes differ from Table S1 in some cases because only samples containing seeds and fruits from trees and shrubs (vs buds and leaves) were used in the mixing model.

* sample consisted of at least 5 individuals combined.

a Calamagrostris arundinacea; Dactylis glomerata; Luzulla pilosa; Mycelis muralis; Veronica sp.

b Aegopodium podagraria; Ajuga reptans; Anemona nemorosa; Dentaria bulbifera; Ficaria verna; Oxalis acetosella; Stellaria holostea.

c Cardamine amara; Chrysosplenium alternifolium; Filipendula ulmaria; Iris pseudacorus; Phragmites communis; Rubus idaeus; Urtica dioica.

The δ^13^C and δ^15^N values of rodent hairs were compared among year categories and species using analysis of variance (ANOVA). We examined how the predicted isotopic values of the diet of the rodent species (Table S3) fit within the vegetation isotope space by first applying diet-hair isotopic discrimination factors (Δ^13^C and Δ^15^N) to our measured rodent hair isotope values. Precise discrimination values are not well understood and are known to vary according to taxonomic group and diet quality [27]. While Miller et al. [28] and De Mots et al. [29] determined Δ^13^C discrimination value of +0.3‰ and a Δ^15^N of +3.3‰ for *Peromyscus*, these values did not provide dietary values falling within the expected isotope foodweb space for our study area primarily due to inappropriately low values of Δ^13^C. So, we instead used a Δ^13^C discrimination factor of +3.2‰ associated with pure herbivores derived by Sponeheimer et al. [30], as summarizes in Table S2. Larger Δ^13^C have similarly been found for voles raised in captivity on experimental diets differing in protein content by Sare et al. [31]. We did not measure protein content of potential foods for the two rodent species in our study site, but note that the %N composition of all plants measured ranged between 0.2 to 7.0%. While we cannot be sure of precise isotopic discrimination factors corresponding to the voles in our wild population, the excellent fit of our rodent hair isotope values within the convex mixing polygon (Figure 1) gives us confidence that the values we have used are reasonable. In addition, regardless of discrimination factors used, the functional response of these rodents to changes in mast abundance in bivariate isotopic space was of primary interest.

## Results

We recorded a broad range of isotope values of potential plant species and tree seeds in the Białowieża Forest (Table S1), which converged into eight isotopically distinct groups (Table 1). After applying appropriate isotopic discrimination factors linking hair to diet, each species fell within a convex polygon in bivariate (δ^13^C, δ^15^N) dietary space (Figure 1), suggesting that our choice of potential dietary items encompassed those actually used. Both species were located primarily along a trajectory between the two strong dietary candidates including seeds of oak, hornbeam, lime and Norway spruce at one end and herbs associated with deciduous forest and graminoids at the other (Figure 2). Assuming only a two-source, two isotope mixing model to derive potential use of deciduous forest herbs vs. mast, we found that while mice depended largely on mast throughout the period of investigation, voles shifted from herbs to mast in mast years (Table 2, Figure 2).

**Table 2:** Predicted (mean and 95% probability) contributions of mast (from oak, hornbeam, lime, spruce isotopic complex) to the diets of the yellow-necked mouse and bank vole through masting cycles assuming only a (SIAR) two-source (deciduous forest herbs vs. mast) mixing model corresponding to the depiction in Figure 2.

	Year		
Species	Mast	Post-mast	Intermediate
Yellow-necked mouse	0.90 (0.64–1.0)	0.97 (0.70–1.0)	0.80 (0.59–0.99)
Bank vole	0.74 (0.55–0.91)	0.54 (0.29–0.88)	0.34 (0.14–0.53)

Hair δ^15^N values for bank voles were significantly different among mast, post-mast and intermediate years (F_2,66_ = 8.18, p<0.001, Figure 2, Table S3). However, the δ^13^C value of vole hairs did not vary in relation to mast events (F_2,65_ = 1.20, p = 0.308, Figure 2, Table S3). The isotopic diet of the yellow-necked mice did not change significantly among years of different seed crop (δ^13^C: F_2,38_ = 1.23, p = 0.304; δ^15^N: F_2,38_ = 0.22, p = 0.807) and was strongly associated with a vegetation group that involved mast in all years (Tables 2, S2). In general, bank vole hairs had lower δ^13^C values (F_1,107_ = 6.55, p<0.05) but higher δ^15^N values (F_1,108_ = 11.67, p<0.001) than those of yellow-necked mice (Figure 2, Table S3).

## Discussion

This study provides exceptional quantitative data on the different dietary response of two consumers to resource pulses. The isotopic dietary niche of both rodents did not overlap in post-mast and intermediate years; however, in mast years, their diets clearly converged and showed isotope values consistent with those of deciduous mast. The yellow-necked mouse diet was generally consistent with one mainly of seeds and did not vary among years. However, the bank vole apparently switched its diet according to food availability and consumed primarily herbs in non-mast years. Previous conventional dietary studies indicate that mice species are fundamentally seed-eaters, while voles mainly consume herbs [32]–[34]. Interestingly, the bank vole has been shown to occupy an intermediate position between mice and other vole species [34], supporting its generalist character shown in this study.

Stable isotope analysis allowed us to obtain direct quantitative evidence for dietary convergence between the rodent species during mast events. However, isotopic similarity between oak, hornbeam, lime and Norway spruce did not allow us to distinguish among these masting trees. While the Bayesian mixing models will always fit a solution to the input data, we note that such models become more informative when coupled with other dietary information that can constrain the range of possible solutions. In our case, it would be particularly interesting to know how subtle isotopic changes in rodent tissues measured through masting cycles translate into actual dietary shifts. Potentially, other stable isotopes (δ^34^S, δ^2^H) or forensic markers (e.g. compound specific isotopic markers) will allow hornbeam and oak inputs to diets of small mammals to be resolved in future studies. Our study provides additional evidence to previous research on rodent population dynamics suggesting that oak and hornbeam masting trigger rodent outbreaks in Białowieża Forest [5], [6], [23] Our study highlights the potential of stable isotopes in the study of resource pulses. Stable isotopes have been used to investigate the long-term use of hoarded pulsed resources [20], the trophic shift of pulse consumers [35] or the influence of marine [18], [36] and terrestrial subsidies [15] on animal populations. However, they have been rarely used to provide empirical evidence of resource pulse use in studies dealing with numerical responses of primary and secondary pulse consumers [18]. Our results well complement the findings of the long-term research on rodent fluctuations in Białowieża Forest [5], [6], [23] and may be inspiring for further research on mast-driven rodent population dynamics and their consequences in the ecosystem. The present study also reveals the potential of faeces and pellets of predators as a source of samples for isotope analysis, of special interest when covering long periods of time.

Although masting is widely recognised as the main factor triggering population outbreaks in mast consumers in temperate deciduous forests [1], [2], [5]–[8], [23], factors associated with the population crash are still debatable [9], [37]. In fact, food competition has been pinpointed as one of the mechanisms acting in a density-dependent way in rodent population dynamics in Białowieża Forest [23]. Our results show that the two species showed a different functional response to the seed pulse, providing insights into how resource fluctuations may influence competitive interactions. Winter reproduction occurs in both species just following the mast, which indicates low intra- and interspecific competition in that period [5]. In the post-mast year, which coincides with the maximum densities of rodents and the beginning of the population crash [5], the species clearly segregate. It can be hypothesized that during periods of food shortage the most dominant species, the yellow-necked mouse [24], likely monopolise mast, the most valuable food, forcing the bank vole to exploit other food resources and diversify its dietary niche. Mice may experience stronger variations in intraspecific competition, which may also be highest during shortage periods (post-mast years). Bank voles may consume mast only in periods of high availability, when foraging costs, including those associated with the presence of competitors, are low. Although their numerical response is similar, fluctuations in vole numbers are much higher than in yellow-necked mice [5], [6], [23] suggesting also that the numerical response to the pulse of the generalist species was stronger. In summary, our results demonstrate that stable isotope analysis are a reliable approach to track nutrient flow in pulsed resources scenarios and encourage to investigate the role that competitive interactions, mediated by resource pulses, may play in rodent fluctuations in forest dominated by mast-producing trees.

## Supporting Information

Table S1 Summary isotope data (mean±S.E.) of Białowieża Forest vegetation samples analyzed in this study, 2007–2011 (N = 161). When sample size is <3, original values are given.
(DOC)

Table S2 Predicted mean contributions of vegetation groups to diets of yellow-necked mice and bank voles based on SIAR mixing model corresponding to the convex polygon shown in figure 1. For illustrative purposes, to demonstrate the largely non informative nature of the mixing model whereby consumer tissues data fall generally centrally within the mixing bivariate spec, we only present results of overall means using the *siarsolomcmcv4* command in SIAR. Vegetation endpoints are: 1. Coniferous forest ground vegetation, 2. Deciduous forest ground vegetation, 3. *Vaccinium* sp., 4. *Pinus sylvestris* seeds, 5. *Corylus avellana* seeds, 6. *Quercus robur*/*Carpinus betulus*/*Tilia cordata*/*Picea abies* seeds, 7. Mushrooms.
(DOC)

Table S3 Stable-carbon and nitrogen isotope values (mean±S.E.) of hairs from yellow-necked mice and bank voles in years of different seed crop, and predicted diet isotopic composition after applying diet-hair discrimination factors (see main text).
(DOC)

## References

[1] Ostfeld RS, Keesing F (2000) Pulsed resources and community dynamics of consumers in terrestrial ecosystems. Trends Ecol Evol 15: 232–237. 10802548 10.1016/s0169-5347(00)01862-0

[2] Yang LH, Bastow JL, Spence KO, Wright AN (2008) What can we learn from resource pulses? Ecology 89: 621–634. 18459327 10.1890/07-0175.1

[3] Yang LH, Edwards KF, Byrnes JE, Bastow JL, Wright AN, et al (2010) A meta-analysis of resource pulse-consumer interactions. Ecol Monogr 80: 125–151.

[4] Kelly D, Sork VL (2002) Mast seeding in perennial plants: Why, how, where? Annu Rev Ecol Syst 33: 427–447.

[5] Pucek Z, Jędrzejewski W, Jędrzejewska B, Pucek M (1993) Rodent population dynamics in a primeval deciduous forest (Białowieża National Park) in relation to weather, seed crop, and predation. Acta Theriol 38: 199–232.

[6] Jędrzejewska B, Jędrzejewski W (1998) Predation in vertebrate communities. The Białowieża Primeval Forest as a case study. Berlin: Springer-Verlag. 450 p.

[7] McShea WJ (2000) The influence of acorn crops on annual variation in rodent and bird populations. Ecology 81: 228–238.

[8] Schmidt KA, Rush SA, Ostfeld RS (2008) Wood thrush nest success and post-fledging survival across a temporal pulse of small mammal abundance in an oak forest. J Anim Ecol 77: 830–837. 18355240 10.1111/j.1365-2656.2008.01378.x

[9] Pedersen AB, Greives TJ (2008) The interaction of parasites and resources cause crashes in a wild mouse population. J Anim Ecol 77: 370–377. 18028357 10.1111/j.1365-2656.2007.01321.x

[10] Previtali MA, Lima M, Meserve PL, Kelt DA, Gutierrez JR (2009) Population dynamics of two sympatric rodents in a variable environment: rainfall, resource availability, and predation. Ecology 90: 1996–2006. 19694146 10.1890/08-0405.1

[11] Hansen TF, Stenseth NC, Henttonen H, Tast J (1999) Interspecific and intraspecific competition as causes of direct and delayed density dependence in a fluctuating vole population. Proc Nat Acad Sci USA 96: 986–991. 9927680 10.1073/pnas.96.3.986 PMC15337

[12] Eccard JA, Ylonen H (2003) Interspecific competition in small rodents: from populations to individuals. Evol Ecol 17: 423–440.

[13] Schoener TW (1974) Resource partitioning in ecological communities. Science 185: 27–39. 17779277 10.1126/science.185.4145.27

[14] Pianka ER (1976) Competition and niche theory. In: May R, editor. Theoretical ecology, principles and applications. Oxford: Blackwell Scientific Publications. 114–141.

[15] Willson JD, Winne CT, Pilgrim MA, Romanek CS, Gibbons JW (2010) Seasonal variation in terrestrial resource subsidies influences trophic niche width and overlap in two aquatic snake species: a stable isotope approach. Oikos 119: 1161–1171.

[16] Dhondt AA (2012) Interspecific competition in birds. New York: Oxford University Press. 296 p.

[17] Wiens JA (1993) Fat times, lean times and competition among predators. Trends Ecol Evol 8: 348–349. 21236192 10.1016/0169-5347(93)90216-C

[18] Stapp P, Polis GA (2003) Influence of pulsed resources and marine subsidies on insular rodent populations. Oikos 102: 111–123.

[19] Duffy DC, Jackson S (1986) Diet studies of seabirds: a review of methods. Colonial Waterbirds 9: 1–17.

[20] Samelius G, Alisauskas RT, Hobson KA, Lariviere S (2007) Prolonging the arctic pulse: long-term exploitation of cached eggs by arctic foxes when lemmings are scarce. J Anim Ecol 76: 873–880. 17714265 10.1111/j.1365-2656.2007.01278.x

[21] Wolf N, Carleton SA, del Rio CM (2009) Ten years of experimental animal isotopic ecology. Funct Ecol 23: 17–26.

[22] Newsome SD, del Rio CM, Bearhop S, Phillips DL (2007) A niche for isotopic ecology. Front Ecol Environ 5: 429–436.

[23] Stenseth NC, Viljugrein H, Jędrzejewski W, Mysterud A, Pucek Z (2002) Population dynamics of *Clethrionomys glareolus* and *Apodemus flavicollis*: seasonal components of density dependence and density independence. Acta Theriol 47: 39–67.

[24] Andrzejewski R, Olszewski J (1963) Social behaviour and interspecific relations in *Apodemus flavicollis* (Melchior, 1834) and *Clethrionomys glareolus* (Schreber, 1780). Acta Theriol 7: 155–168.

[25] Zalewski A (2007) Does size dimorphism reduce competition between sexes? The diet of male and female pine marten at local and wider geographical scales. Acta Theriol 52: 237–250.

[26] Parnell AC, Inger R, Bearhop S, Jackson AL (2010) Source partitioning using stable isotopes: coping with too much variation. PLoS ONE 5(3): e9672. 20300637 10.1371/journal.pone.0009672 PMC2837382

[27] Robbins CT, Felicetti LA, Sponheimer M (2005) The effect of dietary protein quality on nitrogen isotope discrimination. Oecologia 144: 534–540. 15800751 10.1007/s00442-005-0021-8

[28] Miller JF, Millar JS, Longstaffe FJ (2008) Carbon- and nitrogen-isotope tissue-diet discrimination and turnover rates in deer mice (*Peromyscus maniculatus*). Can J Zool 86: 685–691.

[29] De Mots RL, Novak JM, Gaines KF, Gregor AJ, Romanek CS, et al (2010) Tissue-diet discrimination factors and turnover of stable carbon and nitrogen isotopes in white-footed mice (*Peromyscus leucopus*). Can J Zool 88: 961–967.

[30] Sponheimer M, Robinson T, Ayliffe L, Passey B, Roeder B, et al (2003) An experimental study of carbon-isotope fractionation between diet, hair and feces of mammalian herbivores. Can J Zool 81: 871–876.

[31] Sare DTJ, Miller JS, Longstaffe FJ (2005) Tracing dietary protein in red-backed voles (*Clethrionymes gapperi*) using stable isotopes of nitrogen and carbon. Can J Zool 83: 717–725.

[32] Gębczyńska Z (1976) Food habits of the bank vole and phenological phases of plants in an oak hornbeam forest. Acta Theriol 21: 223–236.

[33] Hansson L (1985) The food of bank voles, and wood mice and yellow-necked mice. Sym Zool S 55: 141–168.

[34] Butet A, Delettre YR (2011) Diet differentiation between European arvicoline and murine rodents. Acta Theriol 56: 297–304.

[35] Shaner P-JL, Macko SA (2011) Trophic shifts of a generalist consumer in response to resource pulses. PLoS ONE 6(3): e17970. 21437248 10.1371/journal.pone.0017970 PMC3060883

[36] Spiller DA, Piovia-Scott J, Wright AN, Yang LH, Takimoto G, et al (2010) Marine subsidies have multiple effects on coastal food webs. Ecology 91: 1424–1434. 20503874 10.1890/09-0715.1

[37] Massey FP, Smith MJ, Lambin X, Hartley SE (2008) Are silica defences in grasses driving vole population cycles? Biol Lett 4: 419–422. 18482904 10.1098/rsbl.2008.0106 PMC2474966

