# Supplementary material for: Mast Pulses Shape Trophic Interactions between Fluctuating Rodent Populations in a Primeval Forest
Source: PLoS One. 2012 Dec 10;7(12):e51267. doi: 10.1371/journal.pone.0051267 (PMC3519590; doi:10.1371/journal.pone.0051267)
Supplement: Table S1 — Summary isotope data (mean±S.E.) of Białowieża Forest vegetation samples analyzed in this study, 2007–2011 (N = 161). When sample size is <3, original values are given. (DOC) [file pone.0051267.s001.doc]

**Table S1. Summary isotope data (mean ± S.E.) of Białowieża Forest vegetation samples analyzed in this study, 2007-2011 (N= 161). When sample size is <3, original values are given.**

| **Species** | **N** | **δ^15^N (‰)** | **δ^13^C (‰)** | |
| --- | --- | --- | --- | --- |
| **Ground vegetation: coniferous-dominated forest** | | | | |
| *Calamagrostris arundinacea** | 1 | -5.3 | | -28.4 |
| *Dactylis glomerata** | 1 | -5.2 | | -29.2 |
| *Luzulla pilosa** | 1 | -3.7 | | -30.6 |
| *Mycelis muralis** | 1 | -5.2 | | -29.9 |
| *Veronica* sp.* | 1 | -5.5 | | -30.1 |
| *Vaccinium* sp. | 6 | -5.7±0.5 | | -32.2±0.7 |
| **Ground vegetation: deciduous-dominated forest** | | | | |
| *Aegopodium podagraria** | 1 | -1.9 | | -29.3 |
| *Ajuga reptans** | 1 | -4.7 | | -33.4 |
| *Anemona nemorosa** | 1 | 0.1 | | -28.6 |
| *Dentaria bulbifera** | 1 | -0.5 | | -27.3 |
| *Ficaria verna** | 1 | -1.8 | | -30.2 |
| *Oxalis acetosella** | 1 | -2.3 | | -29.3 |
| *Stellaria holostea** | 1 | -2.5 | | -30.0 |
| **Ground vegetation: deciduous-alderwoods** | | | | |
| *Cardamine amara** | 1 | 5.9 | | -29.0 |
| *Chrysosplenium alternifolium** | 1 | -1.9 | | -28.9 |
| *Filipendula ulmaria** | 1 | 2.8 | | -29.8 |
| *Iris pseudacorus** | 1 | 3.4 | | -30.0 |
| *Phragmites communis** | 1 | 2.6 | | -27.2 |
| *Rubus idaeus** | 1 | -1.9 | | -29.9 |
| *Urtica dioica* | 2 | -1.4,-0.1 | | -27.8,-33.1 |
| **Ground vegetation: meadows** | | | | |
| Graminoids | 8 | -0.1±0.6 | | -30.1±0.7 |
| **Trees in deciduous-dominated forest** | | | | |
| *Acer platanoides* | 10 | -1.8±0.7 | | -30.2±0.7 |
| *Carpinus betulus* | 12 | -1.3±0.8 | | -28.2±0.2 |
| *Corylus avellana* | 10 | -0.6±0.4 | | -32.6±0.6 |
| *Quercus robur* | 12 | -1.7±0.6 | | -27.3±0.3 |
| *Tilia cordata* | 10 | -1.9±0.3 | | -30.9±0.7 |
| **Trees in conifer-dominated forest** | | | | |
| *Picea abies* | 10 | -2.3±0.9 | | -28.2±0.8 |
| *Pinus sylvestris* | 10 | -5.4±1.2 | | -29.0±0.6 |
| **Trees in alderwoods** | | | | |
| *Alnus glutinosa* | 10 | -1.5±0.3 | | -30.3±0.7 |
| *Fraxinus excelsior* | 10 | 0.1±0.5 | | -29.3±0.8 |
| **Other tree species** | | | | |
| *Betula pendula* | 6 | -0.1±1.3 | | -27.6±0.6 |
| *Populus tremula* | 5 | -1.2±0.9 | | -29.1±0.5 |
| *Malus* sp. | 2 | 4.2, 2.3 | | -28.5,-30.1 |
| *Prunus padus* | 1 | -0.6 | | -27.3 |
| *Salix* sp. | 1 | 4.1 | | -28.6 |
| *Sorbus aucuparia* | 5 | -1.4±0.6 | | -33.1±0.7 |
| **Shrubs** |  |  | |  |
| *Euonymus* sp. | 3 | 0±0.8 | | -31.3±0.8 |
| *Ribes* sp. | 1 | -1.1 | | -27.8 |
| **Other groups** |  |  | |  |
| Ferns* | 1 | -0.7 | | -30.8 |
| Mushrooms | 6 | -0.2±0.7 | | -22.2±0.3 |
| Lichens* | 1 | -12.0 | | -26.0 |
| Mosses* | 1 | -5.0 | | -32.1 |

*sample consisted of at least 5 individuals combined.
