# Supplementary material for: Mast Pulses Shape Trophic Interactions between Fluctuating Rodent Populations in a Primeval Forest
Source: PLoS One. 2012 Dec 10;7(12):e51267. doi: 10.1371/journal.pone.0051267 (PMC3519590; doi:10.1371/journal.pone.0051267)
Supplement: Table S2 — Predicted mean contributions of vegetation groups to diets of yellow-necked mice and bank voles based on SIAR mixing model corresponding to the convex polygon shown in figure 1. For illustrative purposes, to demonstrate the largely non informative nature of the mixing model whereby consumer tissues data fall generally centrally within the mixing bivariate spec, we only present results of overall means using the siarsolomcmcv4 command in SIAR. Vegetation endpoints are: 1. Coniferous forest ground vegetation, 2. Deciduous forest ground vegetation, 3. Vaccinium sp., 4. Pinus sylvestris seeds, 5. Corylus avellana seeds, 6. Quercus robur/Carpinus betulus/Tilia cordata/Picea abies seeds, 7. Mushrooms. (DOC) [file pone.0051267.s002.doc]

Table S2. Predicted mean contributions of vegetation groups to diets of yellow-necked mice and bank voles based on SIAR mixing model corresponding to the convex polygon shown in figure 1. For illustrative purposes, to demonstrate the largely non informative nature of the mixing model whereby consumer tissues data fall generally centrally within the mixing bivariate spec, we only present results of overall means using the *siarsolomcmcv4* command in SIAR. Vegetation endpoints are: 1. Coniferous forest ground vegetation, 2. Deciduous forest ground vegetation, 3. *Vaccinium* sp., 4. *Pinus sylvestris* seeds, 5. *Corylus avellana* seeds, 6. *Quercus robur*/ *Carpinus betulus*/ *Tilia cordata*/ *Picea abies* seeds, 7. Mushrooms.

| Species | Vegetation endpoint | | | | | | |
| --- | --- | --- | --- | --- | --- | --- | --- |
|  | 1 | 2 | 3 | 4 | 5 | 6 | 7 |
| Yellow-necked mouse | 0.13 | 0.14 | 0.10 | 0.14 | 0.11 | 0.16 | 0.21 |
| Bank vole | 0.08 | 0.24 | 0.08 | 0.09 | 0.19 | 0.17 | 0.16 |
