# Supplementary material for: Mast Pulses Shape Trophic Interactions between Fluctuating Rodent Populations in a Primeval Forest
Source: PLoS One. 2012 Dec 10;7(12):e51267. doi: 10.1371/journal.pone.0051267 (PMC3519590; doi:10.1371/journal.pone.0051267)
Supplement: Table S3 — Stable-carbon and nitrogen isotope values (mean±S.E.) of hairs from yellow-necked mice and bank voles in years of different seed crop, and predicted diet isotopic composition after applying diet-hair discrimination factors (see main text). (DOC) [file pone.0051267.s003.doc]

Table S3. Stable-carbon and nitrogen isotope values (mean ± S.E.) of hairs from yellow-necked mice and bank voles in years of different seed crop, and predicted diet isotopic composition after applying diet-hair discrimination factors (see main text).

| Year category | N | Hair  δ^13^C (‰) | Predicted diet  δ^13^C (‰) | Hair  δ^15^N (‰) | Predicted diet  δ^15^N (‰) |
| --- | --- | --- | --- | --- | --- |
| **Yellow-necked mouse** | | | | | |
| Mast | 8 | -24.4±0.33 | -27.6±0.33 | 1.2±0.46 | -2.1±0.46 |
| Post-mast | 10 | -23.8±0.46 | -27.0±0.46 | 0.9±0.34 | -2.4±0.34 |
| Intermediate | 23 | -25.0±0.52 | -28.2±0.52 | 1.2±0.25 | -2.1±0.25 |
| **Bank vole** | | | | | |
| Mast | 14 | -25.3±0.20 | -28.5±0.20 | 0.9±0.25 | -2.4±0.25 |
| Post-mast | 17 | -25.5±0.57 | -28.7±0.57 | 1.9±0.39 | -1.4±0.39 |
| Intermediate | 38 | -26.5±0.60 | -29.7±0.60 | 2.6±0.23 | -1.3±0.23 |

Note: One sample of bank vole hairs from mast years did not yield results for δ^13^C.
